# Effective management of acute hemorrhage using an SL One^®^ rapid infusion device in a pediatric patient undergoing nephrectomy for Wilms tumor with inferior vena cava extension: a case report

**DOI:** 10.1186/s40981-025-00788-7

**Published:** 2025-05-04

**Authors:** Shoko Fujioka, Yusuke Miyazaki, Hinako Furuya, Chika Miyazaki, Nobuyuki Katori, Yoshie Taniguchi

**Affiliations:** Department of Anesthesiology, The Jikei University School of Medicine, Tokyo, Japan

**Keywords:** SL One^®^, Rapid infusion device, Hemorrhage, Child, Wilms tumor

## Abstract

**Background:**

Wilms tumor is the most common pediatric renal tumor. Tumor extension into the inferior vena cava (IVC) can increase hemorrhage risk during surgical resection, necessitating rapid transfusion. Pediatric patients have lower circulating blood volume, heightening their susceptibility to hemodynamic instability.

**Case presentation:**

A 2-year-old boy with an IVC-extending Wilms tumor underwent nephrectomy. Anticipating hemorrhage, we employed an SL One^®^ rapid infusion device via a Broviac™ central venous catheter. During a sudden, high-volume bleeding, transfusion was initiated at 23 mL/min and intermittently increased to 150 mL/min while preload was evaluated using transesophageal echocardiography, rapidly stabilizing hemodynamics. No rapid-transfusion-related complications, such as hyperkalemia or hypothermia, were observed, and the postoperative course was uneventful.

**Conclusions:**

In this pediatric case at high risk for acute blood loss, the SL One^®^ provided effective circulatory stabilization without adverse events. Further studies are needed to validate the safety of the SL One^®^ in pediatric patients.

## Background

Wilms tumor, the most common renal tumor in children [1], exhibits a reported annual incidence of 4.3 cases per million population in East Asia, lower than in North America or Europe [2]. Intravascular extension occurs in 4.9–11.8% of cases, with extension into the retrohepatic inferior vena cava (IVC) reported in approximately 0.2–3.2% of cases [3–7]. Intravascular extension significantly increases the risk of surgical complications, including hemorrhage [8, 9]. Consequently, it is crucial to employ an anesthetic management strategy that can rapidly respond to acute hemorrhage in order to maintain cardiac output. This is especially vital in pediatric patients, whose circulating blood volume is smaller than that of adults [10], necessitating thorough preparation.

In the present case, we prepared an SL One^®^ (IMI Co., Ltd., Saitama, Japan) rapid infusion device in advance and utilized it during anesthetic management for resecting a Wilms tumor extending into the IVC. Given the anticipated need for rapid, high-volume transfusion in a pediatric setting, we describe our experience and discuss the potential benefits of employing the SL One^®^ in such cases. This case report follows the Anaesthesia Case Report checklist [11].

## Case presentation

**Fig. 1:**
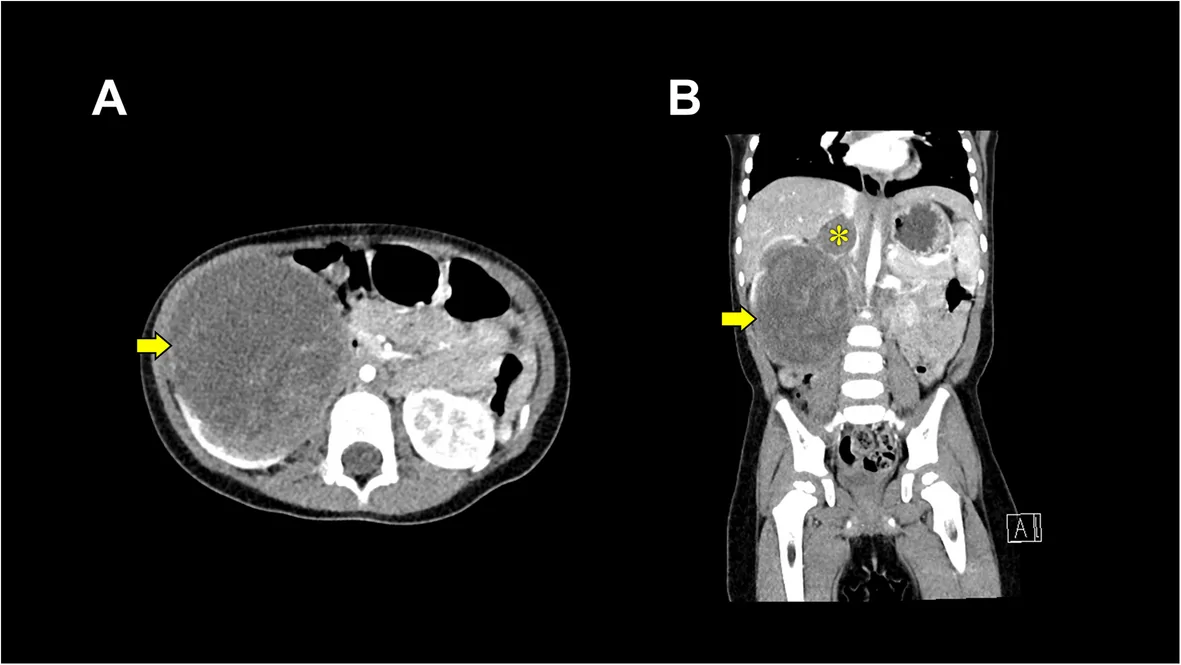
Contrast-enhanced computed tomography of the abdomen. **A** Axial section showing the right renal tumor (yellow arrow). **B** Coronal section illustrating the same tumor (yellow arrow) and its intravascular portion extending into the inferior vena cava (IVC), marked with an asterisk

**Fig. 2:**
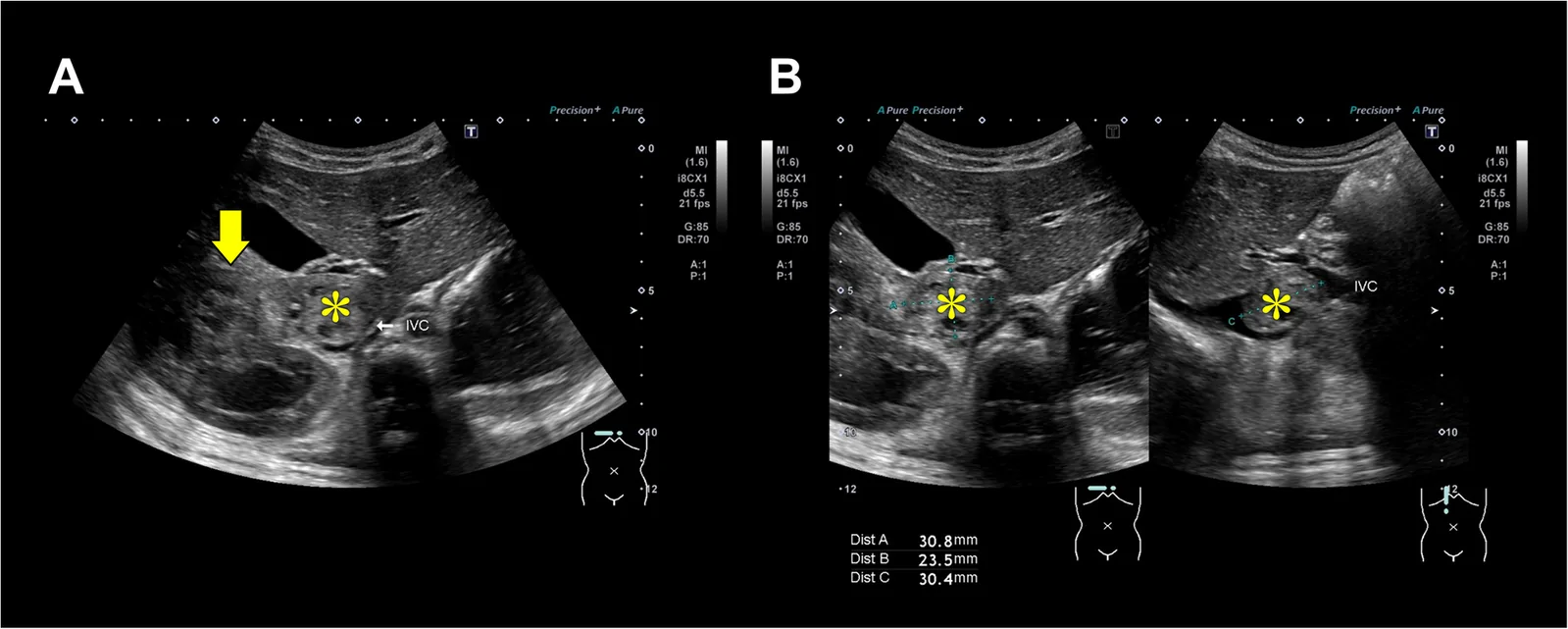
Abdominal ultrasonography. **A** Image demonstrating the right renal tumor (yellow arrow) and its intravascular extension into the inferior vena cava (IVC), marked with an asterisk. **B** Transverse (left) and longitudinal (right) ultrasonographic views of the tumor extending into the IVC, with the intravascular portion marked by an asterisk

A 2-year-10-month-old boy (height 91 cm, body weight 15.5 kg) was brought to a local clinic when his mother noticed a mass in his right abdomen. Although there were no abnormalities in his birth history or developmental milestones, he was admitted to our hospital for further evaluation. Detailed examinations revealed the right renal mass measuring approximately 10 cm in its largest dimension, extending into the IVC to just below the hepatic vein confluence, confirming a diagnosis of Wilms tumor (Figs. 1 and 2). He exhibited no associated congenital anomalies, such as aniridia or genitourinary abnormalities and had no known family history of Wilms tumor. Because the tumor compressed the kidney, renovascular hypertension developed and was managed with amlodipine. Planned treatment involved open right nephrectomy along with removal of the IVC extension. In anticipation of postoperative chemotherapy, placement of a Broviac™ central venous catheter (Becton, Dickinson and Company, Franklin Lakes, NJ, USA) was also planned after the procedure.

Anticipating hemorrhage during resection of the IVC-extending tumor, a multidisciplinary meeting was convened among pediatric and vascular surgeons, pediatricians, anesthesiologists, clinical engineers, nurses, and the blood transfusion department. They shared three main strategies: (1) since the tumor was below the hepatic vein confluence, surgery was prioritized over chemotherapy; (2) if peritoneal dissemination or nodal metastases were found upon laparotomy, tumor resection would be halted in favor of chemotherapy; and (3) with an estimated blood loss exceeding 500 mL, a Broviac™ catheter would be placed following anesthetic induction—prior to tumor resection—and connected to an SL One^®^ (primed with 5% albumin and packed red blood cells [PRBCs]) for rapid transfusion.

The patient was monitored using pulse oximetry, electrocardiography, invasive arterial pressure monitoring, quantitative neuromuscular monitoring, and body temperature measurements. After induction with nitrous oxide and sevoflurane, midazolam 2 mg, fentanyl 75 µg, and rocuronium 20 mg were administered before endotracheal intubation using a McGRATH video laryngoscope (Medtronic, Dublin, Ireland) with a 5.0-mm cuffed endotracheal tube. Following intubation, an epidural catheter was inserted at the T7–8 intervertebral level. Subsequently, pediatric surgeons percutaneously placed a single-lumen, 4.2 Fr Broviac™ catheter via the right subclavian vein. The SL One^®^, primed with 5% albumin and PRBCs, was then connected to the catheter to ensure immediate availability for rapid transfusion if needed. Furthermore, a transesophageal echocardiography (TEE) probe was inserted to evaluate preload, confirm the location of the tumor extending into the IVC, and monitor for possible tumor emboli. Surgery then commenced. When the surgeons attempted to clamp the IVC above the tumor, massive bleeding occurred at the ligated and divided short hepatic vein. In response, transfusion via the SL One^®^ began at 23 mL/min, with the rate intermittently increased as necessary up to 150 mL/min. About 440 mL of blood was lost over 30 min. During roughly the same period, approximately 890 mL of a mixture of 5% albumin and PRBCs was administered from the SL One^®^ reservoir while preload was assessed using TEE, thereby stabilizing the patient’s hemodynamics. Once hemostasis was achieved, the IVC was clamped both above and below the tumor, as well as the left renal vein, and then opened for tumor resection. Because there was no adhesion or invasion, the tumor was readily removed with the right kidney. By the end of surgery, the total blood loss was 480 mL, and urine output was 680 mL. In total, 840 mL of PRBCs, 500 mL of 5% albumin, and 1750 mL of crystalloid were administered, leading to a positive fluid balance of 1930 mL. Notably, the patient’s temperature rose from 36.8 °C at the start of transfusion to 37.4 °C by the end of surgery, confirming that hypothermia never occurred. At that time, the hemoglobin and potassium levels were 14.8 g/dL and 4.6 mmol/L, respectively, and thromboelastography using a TEG^®^ 6s hemostasis analyzer system (Haemonetics, Boston, MA, USA) revealed no abnormality (Table 1). The surgery lasted for 8 h and 30 min, while the anesthesia lasted for 9 h and 48 min. The patient was not extubated in the operating room but was admitted to the pediatric intensive care unit, where he was extubated the following day. No postoperative renal dysfunction was observed, and the patient’s postoperative course was favorable.

**Table 1:** Thromboelastography (TEG^®^ 6s) results from a blood sample taken after tumor resection

Assay	TEG-ACT (sec)	R (min)	K (min)	Angle (°)	A10 (mm)	MA (mm)	LY30 (%)
CK	–	5.7	1.2	74.9	–	57.9	1.4
CRT	106.6	0.6	2.1	67.5	47.6	55.3	1.3
CKH	–	6.8	1.3	73.8	–	55.6	–
CFF	–	–	–	–	17.0	18.0	–

*CK* kaolin assay, *CRT* kaolin and tissue factor assay, *CKH* kaolin assay with heparinase, *CFF* fibrin mesh assay corresponding to fibrinogen concentration. *TEG-ACT* activated clotting time in TEG, *R* reaction time corresponding to the time duration between the initiation of the test and the time at which amplitude of waveform reaches 2 mm, *K* time duration between amplitude at 2 mm and at 20 mm, *Angle* rise angle of the waveform, *A10* amplitude at 10 min after R confirmation, *MA* maximum amplitude, *LY30* amplitude attenuation rate 30 min after MA confirmation

## Discussion

This case involved a 2-year-old boy with Wilms tumor extending into the IVC, who underwent nephrectomy. Anticipating the risk of hemorrhage, a Broviac™ catheter was placed in the right subclavian vein before tumor resection, ensuring readiness for rapid transfusion using the SL One^®^. We believe this strategy enabled effective management of acute hemorrhage over a short period.

In this case, there was a high risk of acute hemorrhage with significant hemodynamic impact for two primary reasons. First, because the tumor extended into the IVC and required IVC incision, the predicted blood loss was estimated to exceed 500 mL. Second, this anticipated blood loss was disproportionately large relative to the estimated circulatory blood volume of approximately 1125 mL in a 2-year-old child weighing about 15 kg [10]. In anticipation of intraoperative hemorrhage, the preoperative meeting was held with all relevant departments to share information and establish a unified treatment approach. As a result of the meeting, the original plan to place the Broviac™ catheter after the right nephrectomy was revised, and the catheter was instead inserted prior to the procedure. Consequently, we were able to utilize a secure central venous line, minimizing the risk of extravasation, and employ the SL One^®^ for immediate high-volume transfusions.

There are two main types of rapid infusion devices: pressure infusion devices and roller pump infusion devices. An example of a pressure infusion device is the Level 1^®^ Fluid Warming System (Smith Medical ASD, Inc., Minneapolis, MN). In contrast, examples of roller pump infusion devices include the SL One^®^ and Belmont^®^ Rapid Infuser RI-2 (Belmont Medical Technologies, Billerica, MA, USA). All three devices share the ability to monitor infusion pressure, warm infused fluids, and detect air bubbles. However, while the Level 1^®^ Fluid Warming System does not automatically remove air bubbles, both the SL One^®^ and the Belmont^®^ Rapid Infuser RI-2 offer automatic bubble removal. Moreover, because roller pump infusion devices facilitate easier flow-rate adjustments, they may be preferable in cases requiring precise control of infusion and transfusion rates. For instance, according to each device’s instruction manual, the Belmont^®^ Rapid Infuser RI-2 has a minimum flow rate of 2.5 mL/min, whereas the SL One^®^ can be set as low as 0.2 mL/min. Consequently, the SL One^®^ may be the most suitable device for finely controlled infusion and transfusion in pediatric patients. Further investigations into its use in pediatric settings are warranted.

Potential complications of massive transfusion using a rapid infusion device include hemolysis, hypothermia, air embolism, and overtransfusion [12–14]. In this case, however, neither hyperkalemia nor hypothermia occurred. Although the hemoglobin level at the end of surgery was 14.8 g/dL—raising concerns about possible overtransfusion of PRBCs—TEE did not reveal any signs of right heart strain during surgery. Likewise, there were no echocardiographic findings suggestive of accidental air infusion associated with the SL One^®^. Taken together, these observations indicate that the SL One^®^ could be used safely in this case.

In this case, the Broviac™ catheter was inserted before surgery to use as a transfusion route via the SL One^®^. A Broviac™ catheter, while less infection-prone than a standard central line due to its subcutaneous tunnel, is also more invasive. In this patient, however, Broviac™ catheter placement was already planned for postoperative chemotherapy. If a tunneled central venous catheter is not indicated, a standard central venous catheter would typically suffice for rapid infusion devices, as peripheral routes risk extravasation during high-volume transfusion [15, 16].

In this 2-year-old patient with Wilms tumor predicted to substantially impact hemodynamics, blood transfusion via the SL One^®^ through a central venous catheter successfully stabilized circulation without complications. These findings suggest that the SL One^®^ may be a feasible option in pediatric cases, although further clinical studies are warranted.

## Data Availability

The data used in this report are available from the corresponding author upon reasonable request.

## Abbreviations

IVC: Inferior vena cava
PRBCs: Packed red blood cells
TEE: Transesophageal echocardiography
